# Susceptibility antibiotic screening reveals high rates of multidrug resistance of *Salmonella, Shigella* and *Campylobacter* in HIV infected and uninfected patients from Mozambique

**DOI:** 10.1186/s12879-023-08219-7

**Published:** 2023-04-21

**Authors:** Delfina F Hlashwayo, Emília V Noormahomed, Leonilde Bahule, Constance A Benson, Robert T Schooley, Betuel Sigaúque, Kim E Barrett, Custódio G Bila

**Affiliations:** 1 Department of Biological Sciences, Faculty of Sciences, Eduardo Mondlane University, Maputo, MZ Mozambique; 2 Department of Animal Health and Epidemiology, Faculty of Veterinary Medicine, Eduardo Mondlane University, Maputo, MZ Mozambique; 3 Department of Microbiology, Faculty of Medicine, Eduardo Mondlane University, Maputo, MZ Mozambique; 4 Department of Medicine, Division of Infectious Diseases and Global Public Health, University of California, San Diego (UCSD), San Diego, US; 5 Mozambique Institute for Health Education and Research (MIHER), Maputo, MZ Mozambique; 6 Manhiça Health Research Center (CISM), Manhiça, MZ Mozambique; 7 Department of Physiology and Membrane Biology, University of California Davis School of Medicine, Sacramento, USA

**Keywords:** Antibiotic resistance, *Salmonella infection and HIV*, *Shigella infection and HIV*, *Campylobacter infection and HIV*, Diarrhea, HIV, Mozambique

## Abstract

**Background:**

Antibacterial resistance is a growing concern worldwide, including in Mozambique. Diarrhea is an important cause of mortality in Mozambique, yet few local studies have reported on the resistance of bacterial pathogens in this context. Therefore, this study aims to characterize antibiotic susceptibility patterns of *Salmonella*, *Shigella* and *Campylobacter* spp. among patients with diarrhea, including those who are HIV-infected and-uninfected.

**Methods:**

We conducted antibiotic susceptibility testing on 157 stool isolates recovered from 129 patients aged between 0 and 80 years with diarrhea, including HIV infected (n = 68) and-uninfected individuals (n = 61), assisted at two health centers in Maputo city. The isolates comprised of 99 *Salmonella*, 45 *Shigella* and 13 *Campylobacter* strains. The Kirby-Bauer disk diffusion method was used on Mueller-Hinton II agar for *Salmonella* and *Shigella* spp., while Mueller-Hinton II agar with 5% defibrinated sheep blood was used for *Campylobacter* spp. We tested six antibiotics listed on the national essential medicines list, including ciprofloxacin, erythromycin, azithromycin, trimethoprim-sulfamethoxazole, gentamicin, and tetracycline.

**Results:**

All isolates were resistant to at least one antibiotic. A high percentage of *Salmonella* spp. isolates were found to be resistant to trimethoprim-sulfamethoxazole (89.9%, n = 89), erythromycin (88.9%, n = 88) and tetracycline (76.8%, n = 76). In addition, 86.6% (n = 39) and 68.9% (n = 31) of *Shigella* isolates were resistant to trimethoprim-sulfamethoxazole and tetracycline, respectively. The majority of *Campylobacter* isolates (92.3%, n = 12) were resistant to erythromycin, azithromycin and tetracycline. Multidrug resistance (MDR) was observed in 79.8% of *Salmonella* spp., 76.9% of *Campylobacter* spp., and 57.8% of *Shigella* spp. Drug susceptibility profiles for *Salmonella* spp. and *Campylobacter* were similar in both HIV-1 infected and uninfected patients. However, *Shigella* spp. isolates obtained from patients without HIV infection were significantly more likely to be resistant to erythromycin, azithromycin or to exhibit multidrug resistance than those obtained from patients with HIV-1 infection (p < 0.05). All *Shigella* spp. and *Campylobacter* spp. isolates were susceptible to gentamicin.

**Conclusion:**

Our study highlights concerning rates of antibiotic resistance and MDR among diarrheal bacterial pathogens in Mozambique. Further research is needed to understand the impact of HIV, ART therapy and immunosuppression on antibiotic resistance. Urgent interventions are essential to prevent the spread of resistant strains.

**Supplementary Information:**

The online version contains supplementary material available at 10.1186/s12879-023-08219-7.

## Background

Mozambique is a developing country in sub-Saharan Africa that faces numerous public health concerns. Major drivers of mortality include HIV/AIDS (25.1%), respiratory infections and tuberculosis (13.8%), cardiovascular diseases (12.0%), neonatal disorders (9.2%), neglected tropical diseases and malaria (8.0%), stroke (6.0%) and enteric infections (3.9%) [1].

Infectious diseases are a significant burden in Mozambique, with diarrhea being one of the most common syndromes [2]. A variety of pathogens are known to cause diarrhea, in the country, including viruses (e.g., rotavirus), parasites (e.g., C*ryptosporidium* spp.), and bacterial agents such as *Escherichia coli* pathotypes, *Shigella* spp., *Salmonella enterica*, *Vibrio cholerae*, *Aeromonas* spp., and *Campylobacter jejuni/coli* [3].

The increasing prevalence of antibiotic resistance in lower- and middle-income countries (LMICs), where the use of antimicrobial drugs is relatively unrestricted, is a growing concern for the impact of bacterial infections in these countries [4]. Recent systematic reviews and meta-analyses reveal a rise in antibiotic resistance over the years [5–9], implying the need for urgent intervention, including more robust antibiotic stewardship programs. In recognition of this pressing public health issue, the World Health Organization has issued a list of bacteria for which new antibiotics are urgently needed, including *Salmonella*, *Shigella* and *Campylobacter* [10] – the three bacteria analyzed in this study.

Study of antibiotic resistance is crucial because it impacts the choice of treatment for infections that require antibiotic therapy. In addition, antibiotic resistance has several implications such as increased morbidity, relapse of infection and more complicated clinical presentations [11]. These consequences can have significant effects on both the patient and the health system.

Amidst the increasing growing global concern about drug resistance [5–9, 11, 12], there is a noticeable paucity of research in Mozambique that investigates the antimicrobial susceptibility patterns of enteric pathogens, especially in the clinical context of diarrhea [13–17].

The lack of information on antibiotic resistance in patients with diarrhea seen at healthcare centers in Maputo city’s suburban and peri-urban areas and across the country is a significant knowledge gap. Furthermore, Mozambique has one of the highest rates of HIV globally (13.2%) [18, 19], yet there is a lack of available information regarding the relationship between HIV infection, viral suppression and antibiotic susceptibility. Therefore, this study aimed to fill this knowledge gap by determining the bacterial susceptibility of *Salmonella*, *Shigella* and *Campylobacter* in both HIV-infected and uninfected patients with diarrhea assisted at two healthcare centers in Maputo city, as well as comparing susceptibility patterns between the two groups and among patients with a HIV-1 low level viraemia and those on virological failure. The study examined susceptibility patterns of six antibiotics included in the national health system’s list of essential medicines advised for antibiotic therapy [20].

## Methods

### Study area and population

The study was conducted in Maputo city, Mozambique, at two healthcare centers located in peri-urban and suburban areas - Centro de Saúde de Mavalane and Centro de Saúde 1° de Maio. These centers were chosen as they are located in densely populated and less urbanized areas, where housing conditions are poor and basic socio-sanitary infrastructure is limited, making the population particularly vulnerable to diarrheal disease [21]. Maputo is the largest city in Mozambique and serves as the country’s administrative, political, economic, and cultural capital. The city has a population of 1,120,867, with females accounting for 577,771 of the total [22]. Additionally, Maputo has the third-highest HIV prevalence rate in the country, at 16.9% [19].

### Patients and bacterial isolation

The study included a total of 129 patients of all ages, both HIV infected (n = 68) and -uninfected (n = 61) who presented with diarrhea at an outpatient consultation and had a positive stool culture for *Salmonella*, *Shigella*, and/or *Campylobacter*. Patients who had received antibiotics in the seven days preceding the consultation, those with undetermined HIV sero-status, and those who had undergone bowel surgery were excluded. The definition of diarrhea used in the study was based on the World Health Organization (WHO) guidelines [23], which define diarrhea as the passing of three or more loose or liquid stools per day, or more frequently than is normal for the individual. The study was conducted between November 2021 and May 2022.

After obtaining informed consent, stool samples were collected within 24 h and transported to the Microbiology laboratory at Faculty of Sciences of Eduardo Mondlane University (UEM) using Cary Blair transport medium.

For the identification of *Salmonella* and *Shigella*, stool samples were cultured in Selenite F Broth at 37 °C for 24 h. The cultures were then subcultured onto *Salmonella-Shigella* agar (Condalab, Spain) for further identification. For *Campylobacter* spp. identification, samples were cultured in Karmali agar enriched with Karmali supplement (Condalab, Spain) under microaerophilic conditions generated by Campygen sachets (Oxoid, UK) at 42 °C for 48 h. Presumptive bacterial identification was based on the colony color and morphology. *Salmonella* seroquick ID kit (SSI Diagnostica, Denmark) was used for agglutination tests to identify *Salmonella typhimurium* and *Salmonella enteritidis.* Furthermore, Gram stain, catalase, hippurate hydrolysis and the H_2_S test were used to identify and differentiate *C. jejuni* and *C. coli* colonies grown in the Karmali agar.

To assess the viral load in HIV infected patients, 4 ml of blood were collected via venipuncture into a tube with Ethylenediaminetetraacetic acid (EDTA) [24]. The blood samples were transported at 4 °C to the Laboratory of Parasitology, Faculty of Medicine, also from UEM for further processing.

RNA was purified from a 0.5 mL plasma sample and the quantitative real-time PCR (qRT-PCR) amplification and detection were done using Abbott RealTime HIV-1 assay in the fully automated m2000rt instrument, as per manufacturer’s instructions (https://www.molecular.abbott/int/en/products/infectious-disease/realtime-hiv-1-viral-load). In a subset of 26 samples without results on the Abbott assay, HIV-1 viral load measurement was performed at the Mavalane General Hospital using the Roche COBAS® AmpliPrep/COBAS® TaqMan® HIV-1 Test, v2.0. RNA was purified from 1 ml plasma aliquots, and detection was performed on the COBAS® 6800 system according to the manufacturer’s instructions (https://diagnostics.roche.com/global/en/products/params/cobas-ampliprep-cobas-taqman-hiv-1-test-v2-0.html#productSpecs).

### Antibiotic susceptibility test

The antibiotic susceptibility testing was performed on the isolates from all 129 patients using six antibiotic disks: ciprofloxacin (CIP) (5 µg), erythromycin (ERY) (15 µg), azithromycin (AZY) (15 µg), trimethoprim-sulfamethoxazole (STX) (25 µg), gentamicin (GEN) (10 µg), and tetracycline (TE) (30 µg). The Kirby–Bauer disk diffusion method was used, and Mueller-Hinton II agar was used for *Salmonella* spp. and *Shigella* spp., while Mueller-Hinton II agar with 5% defibrinated sheep blood was used for *Campylobacter* spp. The inhibition zones were measured, and the isolates were categorized using the Clinical and Laboratory Standards Institute (CLSI) reference Table [25] as R (Resistant), I (Intermediate), or S (Susceptible). We defined organisms as being multidrug resistant if they were not susceptible to at least one agent in three or more antimicrobial classes [26].

### Data analysis

The study data were entered into an Excel 2019 database and analyzed using Epi Info^TM^ version 7.2.5.0 from the Centers for Disease Control and Prevention (CDC). Antibiotic resistance data were presented by dividing the number of resistant isolates by the number of isolates tested. Data were summarized using descriptive statistics. Fisher’s exact test was used to assess the association between HIV infection and the susceptibility pattern of antibiotics (i.e., sensitive, intermediate, or resistant). Additionally, we applied Fisher’s exact test to evaluate the significance of differences in the antibiotic susceptibility profiles of bacterial isolates from HIV-infected patients with viral loads above and below 1000 copies/mL, which indicate a virological failure and low-level viraemia, respectively, according to the WHO guidelines [27]. The Kruskal-Wallis test was utilized to compare the HIV viral load across the three categories of bacterial isolates (sensitive, intermediate, and resistant).[27] A p-value of < 0.05 was considered statistically significant.

## Results

From the stools of 129 patients attending inclusion criteria, a total of 157 isolates were recovered as presented in Table 1. Of the patients included in this study, 89 (69.0%) were female, 26 were children aged 0 to 17 years, and 68 (52.7%) were diagnosed with HIV. The average HIV viral load was 20,413.2 copies/mL, with a minimum of 20 and a maximum of 680,997.0 copies/mL. The dataset of the study can be found in Additional file 1.

**Table 1:** Distribution of *Salmonella*, *Shigella* and *Campylobacter* isolates among the study participants

	Total patients (n = 129)	HIV infected (n = 68)	HIV uninfected (n = 61)
Number of isolates			
*Salmonella* spp.	99	46	53
*Salmonella typhimurium*	5	3	2
*Salmonella enteritidis*	14	6	8
Other *Salmonella*^*a*^	80	37	43
*Shigella* spp.	45	29	16
*Campylobacter* spp.	13	7	6
*Campylobacter coli*	12	7	5
*Campylobacter jejuni*	1	0	1

^a^ Other than *Salmonella typhimurium and Salmonella enteritidis*

### Antibiotic susceptibility profile

The isolates showed a high frequency of resistance to trimethoprim-sulfamethoxazole, erythromycin, and tetracycline (Table 2). The majority of *Salmonella* spp. isolates were resistant to trimethoprim-sulfamethoxazole (89.9%), erythromycin (88.9%), and tetracycline (76.8%). Similarly, the majority of *Shigella* spp. isolates were resistant to trimethoprim-sulfamethoxazole (86.6%) and tetracycline (68.9%). For *Campylobacter* spp., most of the isolates were resistant to erythromycin, azithromycin, and tetracycline (92.3% for all three antibiotics). However, the majority of isolates for all three bacterial species were susceptible to gentamicin, with 97% (n = 96) of *Salmonella* spp. isolates, and 100% of *Shigella* spp. and *Campylobacter* spp. isolates being susceptible to this antibiotic.

**Table 2:** Antibiotic susceptibility profiles of *Salmonella* spp., *Shigella* spp. and *Campylobacter* spp. isolates

Bacterial isolates	n	Pattern	Antibiotic susceptibility, n (%)					
			CIP	ERY	AZY	STX	GEN	TE
*Salmonella* spp.	99	S	66 (66.7)	11 (11.1)	67 (67.7)	7 (7.1)	96 (97.0)	20 (20.2)
		I	27 (27.3)	–	–	3 (3.0)	3 (3.0)	3 (3.0)
		R	6 (6.1)	88 (88.9)	32 (32.3)	89 (89.9)	–	76 (76.8)
*Salmonella typhimurium*	5	S	4 (80.0)	–	5 (100)	–	5 (100)	2 (40.0)
		I	1 (20.0)	–	–	–	–	–
		R	–	5 (100)	–	5 (100)	–	3 (60.0)
*Salmonella enteritidis*	14	S	8 (57.1)	3 (21.4)	10 (71.4)	1 (7.1)	13 (92.9)	3 (21.4)
		I	4 (28.6)	–	–	–	1 (7.1)	1 (7.1)
		R	2 (14.3)	11 (78.6)	4 (28.6)	13 (92.9)	–	10 (71.4)
*Other Salmonella*	80	S	54 (67.5)	8 (10.0)	52 (65.0)	6 (7.5)	78 (97.5)	15 (18.7)
		I	22 (27.5)	–	–	3 (3.7)	2 (2.5)	2 (2.5)
		R	4 (5.0)	72 (90.0)	28 (35.0)	71 (88.8)	–	63 (78.8)
*Shigella* spp.	45	S	27 (60.0)	16 (35.6)	40 (88.9)	3 (6.7)	45 (100)	13 (28.9)
		I	17 (37.8)	–	–	3 (6.7)	–	1 (2.2)
		R	1 (2.2)	29 (64.4)	5 (11.1)	39 (86.6)	–	31 (68.9)
*Campylobacter* spp.	13	S	10 (76.9)	1 (7.7)	1 (7.7)	2 (15.4)	13 (100)	1 (7.7)
		I	–	–	–	2 (15.4)	–	–
		R	3 (23.1)	12 (92.3)	12 (92.3)	9 (69.2)	–	12 (92.3)
*Campylobacter jejuni*	1	S	–	–	–	–	1 (100)	–
		I	–	–	–	–	–	–
		R	1 (100)	1 (100)	1 (100)	1 (100)	–	1 (100)
*Campylobacter coli*	12	S	10 (83.3)	1 (8.3)	1 (8.3)	2 (16.7)	12 (100)	1 (8.3)
		I	–	–	–	2 (16.7)	–	–
		R	2 (16.7)	11 (91.7)	11 (91.7)	8 (66.6)	–	11 (91.7)

Note: S = Sensitive; I = Intermediate; R = Resistant

Multidrug resistance was observed in a high proportion of the isolates, specifically, with 79.8% of *Salmonella* spp., 57.8% of *Shigella* spp. and 76.9% of *Campylobacter* spp. exhibiting resistance to multiple antibiotic classes (Table 3). Most isolates showed resistance to three classes of antibiotics, while only one (1.0%) *Salmonella* spp. isolate was found to be resistant to five classes of antibiotics.

**Table 3:** Proportions of multidrug resistance among *Campylobacter* spp., *Salmonella* spp., and *Shigella* spp. isolates

Bacterial isolates	n	Multidrug resistance to several classes of antibiotics n(%)			
		Total MDR*	3	4	5
*Salmonella* spp.	99	79 (79.8)	53 (53.5)	25 (25.3)	1 (1.0)
*Salmonella typhimurium*	5	4 (80.0)	4 (80.0)	–	–
*Salmonella enteritidis*	14	12 (85.7)	9 (64.3)	3 (21.4)	–
*Other Salmonella*	80	63 (78.8)	40 (50.0)	22 (27.5)	1 (1.3)
*Shigella* spp.	45	26 (57.8)	15 (33.3)	11 (24.4)	–
*Campylobacter* spp.	13	10 (76.9)	7 (53.8)	3 (23.1)	–
*Campylobacter jejuni*	1	1 (100)	–	1 (100)	–
*Campylobacter coli*	12	9 (75.0)	7 (58.3)	2 (16.7)	–

MDR: Multidrug resistant

### Comparison of antibiotic resistance among HIV infected and uninfected patients

Additional file 2 presents the comparison of antibiotic susceptibility and multidrug resistance among HIV infected and uninfected patients, as well as the analysis of association between antibiotic susceptibility profile and HIV viral load.

We did not find any correlation between antibiotic resistance and HIV or viral load for both *Salmonella* and *Campylobacter* spp. (p > 0.05). However, *Shigella* showed significant differences in the susceptibility pattern between HIV infected and uninfected patients, specifically for erythromycin, azithromycin and MDR isolates (p < 0.05) (Tables 6 and 7 of Additional file 2). HIV-infected patients had a lower proportion of *Shigella* isolates resistant to erythromycin (48.3%, n = 14), compared to uninfected patients (93.7%, n = 15). Similarly, compared to uninfected patients, HIV-infected patients had fewer azithromycin-, and MDR isolates of *Shigella* spp. in the study.

No statistically significant differences were observed for the antibiotic susceptibility profiles of three bacteria when comparing viral load using both Fischer’s exact test and Kruskal Wallis test.

## Discussion

In our study, we found that certain antibiotic-resistant strains of *Salmonella*, *Shigella*, and *Campylobacter* spp. were present in patients with diarrhea attended at health facilities in the peri-urban and suburban areas of Maputo city. It is important to note that other agents, such as rotavirus [3], *E. coli* pathotypes, and parasites like *Giardia* spp. [28, 29], may have also contributed to the onset of diarrhea in these patients. Therefore, further cohort studies are needed to identify the primary pathogens associated with diarrhea and their antimicrobial resistance (AMR) patterns across a broad range of enteric pathogens.

Of particular concern is the fact that over 60% of all isolates were resistant to trimethoprim-sulfamethoxazole. This is especially alarming as this antibiotic is often the treatment of choice for patients presenting with diarrhea [30, 31] and other health conditions, and is also used as a prophylaxis for opportunistic infections in people living with HIV [32]. Thus, it may be necessary to explore other treatment options for diarrhea and consider using alternative antibiotics for prophylaxis in individuals with HIV. Even more troubling is the high prevalence of MDR among these isolates which imposes the risk of fewer therapeutic options in clinical cases requiring antibiotic treatment [7–9].

Comparing the six antibiotics tested in this study, gentamicin remains as the antibiotic with most susceptible isolates. Apart from the possible reasons mentioned before, we think that most probably, the route of administration of gentamicin, which is only given intravenously, plays an important role for protecting against the indiscriminate use of antibiotics in informal market or sold in pharmacies.

This study’s findings demonstrate an increase in antimicrobial resistance compared to a study conducted in Manhiça, located in Maputo province, Mozambique [13]. The previous study, conducted from 2000 to 2001, found that 38% of *Salmonella* spp. isolated from children under five years with diarrhea were resistant to trimethoprim-sulfamethoxazole, while in our study, 89.9% of the isolates were resistant to that antibiotic. The same trend was observed for *Campylobacter* spp., with a previous study reporting a 22% tetracycline resistance pattern, while in our study, 92.3% of the isolates were resistant to that antibiotic.

A additional study conducted in Manhiça from 2001 to 2003, focusing on children under five years with diarrhea, found *Shigella* spp. resistance patterns to be similar to our study, with 84% of the isolates resistant to trimethoprim-sulfamethoxazole and 66% resistant to tetracycline. However, the previous study reported lower *Salmonella* spp. resistance patterns, with only 18% resistant to trimethoprim-sulfamethoxazole and 15% resistant to tetracycline [14].

Although our study did not analyze all enteric bacteria in Mozambique, other studies have reported the presence of antibiotic resistance in these bacteria. For instance, a study conducted in 2004, in Beira city, Sofala province, central Mozambique, found that all 32 strains of *Vibrio parahaemolyticus* serovar O3:K6 (n = 32) isolated from patients over two years of age with diarrhea were resistant to ampicillin, but susceptible to other antibiotics such as tetracycline, trimethoprim-sulfamethoxazole, nalidixic acid, furazolidone, erythromycin and ciprofloxacin [15]. Similarly, a study conducted between 2014 and 2017 in four Mozambican provinces found a high resistance to ampicillin (97.8%) in isolates of diarrheal *E. coli* from diarrheic children aged 0–14 years [16].

The most recent study conducted on antibiotic susceptibility of enteric bacteria in Maputo city, which included children below 14 months old from low-income neighborhoods between 2015 and 2017, found that the most commonly detected antibiotic resistance genes in stools conferred resistance to macrolides, lincosamides, streptogramin b (100%); tetracyclines (98%); β-lactams (94%); and aminoglycosides (84%) [17].

Multidrug resistance was found in a previous study conducted in Manhiça from 2001 to 2003, which included children under five years old with diarrhea [14]. According to the study, multidrug resistance was detected in *Salmonella* and *Shigella* at proportions of 23% and 65%, respectively. In contrast, our study detected MDR *Salmonella* and *Shigella* at proportions of 79.8% and 57.7%, respectively, indicating a substantial increase in MDR *Salmonella* occurrence over an interval of more than 19 years. Moreover, a recent study in Maputo city found MDR bacteria in drinking water samples [33], which indicates an environmental contamination that can lead the spread of these bacteria in the community.

Regarding the association between antibiotic susceptibility and HIV, it is important to note that a recent worldwide systematic review and meta-analysis found an increased risk of antimicrobial resistance in HIV infected patients. However, the authors pointed out that more studies are needed to further address this association, as most studies were from either the United States of America (35%) or from South Africa (23%) [34]. The authors suggested that people living with HIV are at increased risk of infections with resistant organisms due to more frequent healthcare utilization, suggesting nosocomial infection or transmission.

We were surprised to find in our study that people without HIV infection were more likely to harbor *Shigella* strains that were resistant to antibiotics, compared to those with HIV infection. One possible explanation for this finding is that people with HIV infection have more access to the healthcare system due to monitoring through periodic consultations, and for that reason medications are more likely to be correctly prescribed than among those without HIV infection who might be accessing antibiotics more frequently without a prescription. However, it will be necessary to evaluate other related variables including the likelihood of exposure to resistant pathogens in both HIV infected and uninfected populations, levels of HIV-1 induced immunodeficiency and the prevalence of antiretroviral therapy intake, to have a broader understanding of the relationships between HIV infection and antibiotic resistance in enteric bacteria. We also acknowledge that cultural practices may play a role in the use of over-the-counter drugs, and this is an important factor to consider in public health interventions aimed at promoting appropriate antibiotic use. Therefore, further research is needed to investigate the impact of cultural practices on antibiotic use, and how these practices may contribute to the emergence and spread of antibiotic resistance.

Overall, the high prevalence of antibiotic resistance findings in this study is likely due to multiple factors, including unrestricted and inappropriate use of antibiotics through self-medication, the sale of antibiotics in informal markets [35, 36], cross-contamination through environmental and food sources [33, 37, 38] and the use of antibiotics in animal husbandry without proper guidance from healthcare professionals [39–42].

Our study, along with other studies from across the African continent [5, 43, 44], highlights the urgent need for effective antimicrobial stewardship and infection control measures to combat the rise of antibiotic resistance. It is crucial to address the misuse and overuse of antibiotics, as well as improve the availability and accessibility of effective antibiotics in low-resource settings. Additionally, interventions such as improved sanitation and hygiene practices can also help reduce the burden of enteric infections and subsequent antibiotic use. Collaborative efforts among healthcare providers, policymakers, and communities are necessary to address this growing threat and ensure the effectiveness of antibiotics for generations to come.

The study had some limitations that should be noted. One such limitation is the lack of information on the patients’ antiretroviral therapeutic regimens, which could have been a variable to analyze, given that the antiretroviral therapy may affect the risk of infection by bacterial pathogens and hospitalizations [34]. Another limitation is the absence of data on the antibiotics prescribed or the outcome of diarrheal disease which could have provided insight into the clinical relevance of our findings, especially with regard to the analysis of treatment failure due to the high proportion of antibiotic resistance.

## Conclusions

The study findings revealed a high prevalence of antibiotic resistant organisms in patients with diarrhea infected with *Salmonella, Shigella* and *Campylobacter*. The resistance to first-line antibiotics, such as trimethoprim-sulfamethoxazole was prevalent, indicating the urgency of implementing effective measures to control antibiotic resistance.

This study’s findings hold significance in aiding clinicians to select appropriate antibiotics for treating patients with diarrhea. Gentamicin was found to have the highest susceptibility rates among the antibiotics tested, with 97% of *Salmonella* and all isolates of *Shigella* and *Campylobacter* being susceptible to it. However, *Shigella* isolates from those without HIV-1 infection exhibited frequently resistance to erythromycin, azithromycin and multidrug resistance. Moreover, viral load did not appear to be associated with antibiotic susceptibility.

Our results highlight the critical need for implementing measures to promote responsible antibiotic use in both human and animal populations. Additionally, reinforcing pharmaceutical inspection and improving antimicrobial resistance testing and reporting is crucial in monitoring and responding to emerging resistance strains.

Future studies should focus on exploring the complex interplay between HIV and antibiotic resistance, taking into account the measurement of both CD4 cell count and viral load, as well as evaluating any potential effects of ART. These studies will be essential in developing effective strategies for preventing and managing antibiotic resistance in people living with HIV.

## Electronic supplementary material

Below is the link to the electronic supplementary material.

Supplementary Material 1

Supplementary Material 2

## Data Availability

All data generated or analysed during this study are included in this published article and its supplementary information files.
