# Supplementary material for: Susceptibility antibiotic screening reveals high rates of multidrug resistance of Salmonella, Shigella and Campylobacter in HIV infected and uninfected patients from Mozambique
Source: BMC Infect Dis. 2023 Apr 21;23:255. doi: 10.1186/s12879-023-08219-7 (PMC10122284; doi:10.1186/s12879-023-08219-7)
Supplement: Supplementary file 2 — Supplementary Material 2 [file 12879_2023_8219_MOESM2_ESM.docx]

| **Susceptibility antibiotic screening reveals high rates of multidrug resistance of *Salmonella*, *Shigella* and *Campylobacter* in HIV infected and uninfected patients from Mozambique** |
| --- |

**Analysis of antibiotic susceptibility stratified by HIV status and viral load**

**Table 1.** Antibiotic susceptibility of *Salmonella* isolates in HIV infected and uninfected patients, with significance of differences determined by Fisher's Exact test.

| **Antibiotic** | **Total Study n(%)** | | | **HIV infected n(%)** | | | **HIV uninfected n(%)** | | | **P-value** |
| --- | --- | --- | --- | --- | --- | --- | --- | --- | --- | --- |
|  | (n=99) | | | (n=46) | | | (n=53) | | |  |
|  | **S** | **I** | **R** | **S** | **I** | **R** | **S** | **I** | **R** |  |
| CIP | 66 (66.7%) | 27 (27.3%) | 6 (6.1%) | 28 (60.9%) | 16 (34.8%) | 2 (4.3%) | 38 (71.7%) | 11 (20.8%) | 4 (7.5%) | 0.303 |
| ERY | 11 (11.1%) | - | 88 (88.9%) | 5 (10.9%) | - | 41 (89.1%) | 6 (11.3%) | - | 47 (88.7%) | 1.000 |
| AZY | 67 (67.7%) | - | 32 (32.3%) | 32 (69.6%) | - | 14 (30.4%) | 35 (66.0%) | - | 18 (34.0%) | 0.830 |
| STX | 7 (7.1%) | 3 (3.0%) | 89 (89.9%) | 5 (10.9%) | 2 (4.3%) | 39 (84.8%) | 2 (3.8%) | 1 (1.9%) | 50 (94.3%) | 0.334 |
| GEN | 96 (97.0%) | 3 (3.0%) | - | 46 (100%) | - | - | 50 (94.3%) | 3 (5.7%) | - | 0.246 |
| TE | 20 (20.2%) | 3 (3.0%) | 76 (76.8%) | 10 (21.7%) | 1 (2.2%) | 35 (76.1%) | 10 (18.9%) | 2 (3.8%) | 41 (77.4%) | 0.921 |

**Table 2.** Multidrug resistance of *Salmonella* isolates in HIV infected and uninfected patients, with significance of differences determined by Fisher's Exact test.

| **Variable** | **Total Study n(%)** | | **HIV infected n(%)** | | **HIV uninfected n(%)** | | **P-value** |
| --- | --- | --- | --- | --- | --- | --- | --- |
|  | (n=99) | | (n=46) | | (n=53) | |  |
| MDR | **Yes** | **No** | **Yes** | **No** | **Yes** | **No** | 1.000 |
|  | 79 (79.8%) | 20 (20.2%) | 37 (80.4%) | 9 (19.6%) | 42 (79.2%) | 11 (20.8%) |  |

**Table 3.** Antibiotic susceptibility profiles of *Salmonella* isolates from HIV infected patients with viral load above and below 1000 copies/ml, with results of Fisher’s Exact test for differences.

| **Antibio**  **tic** | **Total Study n(%)** | | | **>1000 copies/ml n(%)** | | | **<1000 copies/ml n(%)** | | | **P-value** |
| --- | --- | --- | --- | --- | --- | --- | --- | --- | --- | --- |
|  | (n=45) | | | (n=5) | | | (n=40) | | |  |
|  | **S** | **I** | **R** | **S** | **I** | **R** | **S** | **I** | **R** |  |
| CIP | 28 (62.2%) | 15 (33.3%) | 2 (4.4%) | 4 (80.0%) | 1 (20.0%) | - | 24 (60.0%) | 14 (35.0%) | 2 (5.0%) | 0.719 |
| ERY | 5 (11.1%) | - | 40 (88.9%) | 1 (20.0%) | - | 4 (80.0%) | 4 (10.0%) | - | 36 (90.0%) | 0.461 |
| AZY | 31 (68.9%) | - | 14 (31.1%) | 4 (80.0%) | - | 1 (20.0%) | 27 (67.5%) | - | 13 (32.5%) | 1.000 |
| STX | 5 (11.1%) | 2 (4.4%) | 38 (84.4%) | - | - | 5 (100%) | 5 (12.5%) | 2 (5.0%) | 33 (82.5%) | 1.000 |
| GEN | 45 (100.0%) | - | - | 5 (100%) | - | - | 40 (100%) | - | - | - |
| TE | 10 (22.2%) | 1 (2.2%) | 34 (75.6%) | - | - | 5 (100%) | 10 (25.0%) | 1 (2.5%) | 29 (72.5%) | 0.620 |

**Table 4.** Multidrug resistance of *Salmonella* isolates in HIV infected with viral load above and below 1000 copies/ml, with results of Fisher’s Exact test for differences.

| **Variable** | **Total Study n(%)** | | **>1000 copies/ml n(%)** | | **<1000 copies/ml n(%)** | | **P-value** |
| --- | --- | --- | --- | --- | --- | --- | --- |
|  | (n=45) | | (n=5) | | (n=40) | |  |
| MDR | **Yes** | **No** | **Yes** | **No** | **Yes** | **No** | 1.000 |
|  | 36 (80.0%) | 9 (20.0%) | 4 (80.0%) | 1 (20.0%) | 32 (80.0%) | 8 (20.0%) |  |

**Table 5.** Assessment of the association between *Salmonella* antibiotic susceptibility profiles and HIV viral load in infected patients using Kruskal-Wallis test.

| **Variable** | **Categories** | **n** | **HIV Viral load** | | | | **Kruskal-Wallis H** | **P-value** |
| --- | --- | --- | --- | --- | --- | --- | --- | --- |
|  |  |  | **Average** | **SD** | **Min** | **Max** |  |  |
| CIP | S | 28 | 38,197.0 | 141,234.5 | 20 | 680,997.0 | 0.148 | 0.929 |
|  | I | 15 | 291.3 | 765.6 | 20 | 3,000.0 |  |  |
|  | R | 2 | 75 | 0 | 75 | 75 |  |  |
| ERY | S | 5 | 9,403.6 | 20,890.1 | 20 | 46,773.0 | 0.065 | 0.799 |
|  | I | - | - | - | - | - |  |  |
|  | R | 40 | 25,675.5 | 118,849.1 | 20 | 680,997.0 |  |  |
| AZY | S | 31 | 23,757.3 | 122,265.4 | 20 | 680,997.0 | 2.029 | 0.154 |
|  | I | - | - | - | - | - |  |  |
|  | R | 14 | 24,111.4 | 90,014.9 | 20 | 336,859.0 |  |  |
| STX | S | 5 | 42 | 30.1 | 20 | 75 | 3.472 | 0.176 |
|  | I | 2 | 75 | 0 | 75 | 75 |  |  |
|  | R | 38 | 28,254.6 | 121,821.7 | 20 | 680,997.0 |  |  |
| GEN | S | 45 | 23,867.5 | 112,189.1 | 20 | 680,997.0 | - | - |
|  | I | - | - | - | - | - |  |  |
|  | R | - | - | - | - | - |  |  |
| TE | S | 10 | 65.4 | 24.1 | 20 | 82.0 | 2.034 | 0.362 |
|  | I | 1 | 904.0 | N/A | 904.0 | 904.0 |  |  |
|  | R | 34 | 31,543.5 | 128,582.6 | 20 | 680,997.0 |  |  |
| MDR | Yes | 36 | 28,523.0 | 125,123.9 | 20 | 680,997.0 | 0.5701 | 0.450 |
|  | No | 9 | 5,245.3 | 15,572.9 | 20 | 46,773.0 |  |  |

**Table 6.** Antibiotic susceptibility of *Shigella* isolates in HIV infected and uninfected patients, with significance of differences determined by Fisher’s Exact test.

| **Antibio**  **tic** | **Total Study n(%)** | | | **HIV infected n(%)** | | | **HIV uninfected n(%)** | | | **P-value** |
| --- | --- | --- | --- | --- | --- | --- | --- | --- | --- | --- |
|  | (n=45) | | | (n=29) | | | (n=16) | | |  |
|  | **S** | **I** | **R** | **S** | **I** | **R** | **S** | **I** | **R** |  |
| CIP | 27 (60.0%) | 17 (37.8%) | 1 (2.2%) | 17 (58.6%) | 11 (37.9%) | 1 (3.4%) | 10 (62.5%) | 6 (37.5%) | - | 1.000 |
| ERY | 16 (35.6%) | - | 29 (64.4%) | 15 (51.7%) | - | 14 (48.3%) | 1 (6.3%) | - | 15 (93.7%) | **0.003** |
| AZY | 40 (88.9%) | - | 5 (11.1%) | 28 (96.6%) | - | 1 (3.4%) | 12 (75.0%) | - | 4 (25.0%) | **0.047** |
| STX | 3 (6.7%) | 3 (6.7%) | 39 (86.6%) | 3 (10.3%) | 2 (6.9%) | 24 (82.8%) | - | 1 (6.3%) | 15 (93.7%) | 0.790 |
| GEN | 45 (100%) | - | - | 29 (100%) | - | - | 16 (100%) | - | - | 1.000 |
| TE | 13 (28.9%) | 1 (2.2%) | 31 (68.9%) | 9 (31.0%) | 1 (3.5%) | 19 (65.5%) | 4 (25.0%) | - | 12 (75.0%) | 0.832 |

**Table 7.** Multidrug resistance of *Shigella* isolates in HIV infected and uninfected patients, with significance of differences determined by Fisher's Exact test.

| **Variable** | **Total Study n(%)** | | **HIV infected n(%)** | | **HIV uninfected n(%)** | | **P-value** |
| --- | --- | --- | --- | --- | --- | --- | --- |
|  | (n=45) | | (n=29) | | (n=16) | |  |
| MDR | **Yes** | **No** | **Yes** | **No** | **Yes** | **No** | **0.027** |
|  | 26 (57.8%) | 19 (42.2%) | 13 (44.8%) | 16 (55.2%) | 13 (81.3%) | 3 (18.7%) |  |

**Table 8.** Antibiotic susceptibility profiles of *Shigella* isolates in HIV infected patients with viral load above and below 1000 copies/ml, with results of Fisher’s Exact test for differences.

| **Antibiotic** | **Total Study n(%)** | | | **>1000 copies/ml n(%)** | | | **<1000 copies/ml n(%)** | | | **P-value** |
| --- | --- | --- | --- | --- | --- | --- | --- | --- | --- | --- |
|  | (n=29) | | | (n=5) | | | (n=24) | | |  |
|  | **S** | **I** | **R** | **S** | **I** | **R** | **S** | **I** | **R** |  |
| CIP | 17 (58.6%) | 11 (37.9%) | 1 (3.5%) | 4 (80.0%) | 1 (20.0%) | - | 13 (54.2%) | 10 (41.7%) | 1 (4.2%) | 0.685 |
| ERY | 15 (51.7%) | - | 14 (48.3%) | 4 (80.0%) | - | 1 (20.0%) | 11 (45.8%) | - | 13 (54.2%) | 0.330 |
| AZY | 28 (96.6%) | - | 1 (3.4%) | 4 (80.0%) | - | 1 (20.0%) | 24 (100%) | - | - | 0.172 |
| STX | 3 (10.3%) | 2 (6.9%) | 24 (82.8%) | 1 (20.0%) | - | 4 (80.0%) | 2 (8.3%) | 2 (8.3%) | 20 (83.3%) | 0.642 |
| GEN | 29 (100%) | - | - | 5 (100%) | - | - | 24 (100%) | - | - | 1.000 |
| TE | 9 (31.0%) | 1 (3.5%) | 19 (65.5%) | 1 (20.0%) | - | 4 (80.0%) | 8 (33.3%) | 1 (4.2%) | 15 (62.5%) | 1.000 |

**Table 9.** Multidrug resistance of *Shigella* isolates in HIV infected patients with viral load above and below 1000 copies/ml, with results of Fisher’s Exact test for differences.

| **Variable** | **Total Study n(%)** | | **>1000 copies/ml n(%)** | | **<1000 copies/ml n(%)** | | **P-value** |
| --- | --- | --- | --- | --- | --- | --- | --- |
|  | (n=29) | | (n=5) | | (n=24) | |  |
| MDR | **Yes** | **No** | **Yes** | **No** | **Yes** | **No** | 0.343 |
|  | 13 (44.8%) | 16 (55.2%) | 1 (20.0%) | 4 (80.0%) | 12 (50.0%) | 12 (50.0%) |  |

**Table 10.** Assessment of the association between *Shigella* antibiotic susceptibility profiles and HIV viral load in infected patients using Kruskal-Wallis test.

| **Variable** | **Categories** | **n** | **HIV Viral load** | | | | **Kruskal-Wallis H** | **P-value** |
| --- | --- | --- | --- | --- | --- | --- | --- | --- |
|  |  |  | **Average** | **SD** | **Min** | **Max** |  |  |
| CIP | S | 17 | 2,140.8 | 5,089.9 | 20 | 15,840.0 | 2.231 | 0.328 |
|  | I | 11 | 16,226.1 | 53,654.3 | 20 | 178,000.0 |  |  |
|  | R | 1 | 75 | N/A | 75 | 75 |  |  |
| ERY | S | 15 | 2,404.9 | 5,382.7 | 20 | 15,840.0 | 0.001 | 0.982 |
|  | I | - | - | - | - | - |  |  |
|  | R | 14 | 12,777.2 | 47,554.4 | 20 | 178,000.0 |  |  |
| AZY | S | 28 | 1,319.8 | 4,053.7 | 20 | 15,840.0 | 3.081 | 0.079 |
|  | I | - | - | - | - | - |  |  |
|  | R | 1 | 178,000.0 | N/A | 178,000.0 | 178,000.0 |  |  |
| STX | S | 3 | 5,330.0 | 9,101.9 | 75 | 15,840.0 | 1.760 | 0.415 |
|  | I | 2 | 47.5 | 38.9 | 20 | 75 |  |  |
|  | R | 24 | 8,286.3 | 36,282.5 | 20 | 178,000.0 |  |  |
| GEN | S | 29 | 7,412.2 | 33,049.3 | 20 | 178,000.0 | - | - |
|  | I | - | - | - | - | - |  |  |
|  | R | - | - | - | - | - |  |  |
| TE | S | 9 | 1,812.1 | 5,260.6 | 20 | 15,840.0 | 0.221 | 0.895 |
|  | I | 1 | 75 | N/A | 75 | 75 |  |  |
|  | R | 19 | 10,451.1 | 40,722.8 | 20 | 178,000.0 |  |  |
| MDR | Yes | 13 | 13,750.1 | 49,350.9 | 20 | 178,000.0 | 0.305 | 0.581 |
|  | No | 16 | 2,262.8 | 5,231.2 | 20 | 15,840.0 |  |  |

**Table 11.** Antibiotic susceptibility of *Campylobacter* isolates in HIV infected and uninfected patients, with significance of differences determined by Fisher's Exact test.

| **Antibio**  **tic** | **Total Study n(%)** | | | **HIV infected n(%)** | | | **HIV uninfected n(%)** | | | **P-value** |
| --- | --- | --- | --- | --- | --- | --- | --- | --- | --- | --- |
|  | (n=13) | | | (n=7) | | | (n=6) | | |  |
|  | **S** | **I** | **R** | **S** | **I** | **R** | **S** | **I** | **R** |  |
| CIP | 10 (76.9%) | - | 3 (23.1%) | 6 (85.7%) | - | 1 (14.3%) | 4 (66.7%) | - | 2 (33.3%) | 0.559 |
| ERY | 1 (7.7%) | - | 12 (92.3%) | 1 (14.3%) | - | 6 (85.7%) | - | - | 6 (100%) | 1.000 |
| AZY | 1 (7.7%) | - | 12 (92.3%) | 1 (14.3%) | - | 6 (85.7%) | - | - | 6 (100%) | 1.000 |
| STX | 2 (15.4%) | 2 (15.4%) | 9 (69.2%) | 1 (14.3%) | 2 (28.6%) | 4 (57.1%) | 1 (16.7%) | - | 5 (83.3%) | 0.706 |
| GEN | 13 (100%) | - | - | 7 (100%) | - | - | 6 (100%) | - | - | 1.000 |
| TE | 1 (7.7%) |  | 12 (92.3%) | 1 (14.3%) | - | 6 (85.7%) | - | - | 6 (100%) | 1.000 |

**Table 12.** Multidrug resistance of *Campylobacter* isolates in HIV infected and uninfected patients, with significance of differences determined by Fisher's Exact test.

| **Variable** | **Total Study n(%)** | | **HIV infected n(%)** | | **HIV uninfected n(%)** | | **P-value** |
| --- | --- | --- | --- | --- | --- | --- | --- |
|  | (n=13) | | (n=7) | | (n=6) | |  |
| MDR | **Yes** | **No** | **Yes** | **No** | **Yes** | **No** | 1.000 |
|  | 10 (76.9%) | 3 (23.1%) | 5 (71.4%) | 2 (28.6%) | 5 (83.3%) | 1 (16.7%) |  |

**Table 13.** Antibiotic susceptibility profiles of *Campylobacter* isolates in HIV infected patients with viral load above and below 1000 copies/ml, with results of Fisher’s Exact test for differences.

| **Antibiotic** | **Total Study n(%)** | | | **>1000 copies/ml n(%)** | | | **<1000 copies/ml n(%)** | | | **P-value** |
| --- | --- | --- | --- | --- | --- | --- | --- | --- | --- | --- |
|  | (n=7) | | | (n=1) | | | (n=6) | | |  |
|  | **S** | **I** | **R** | **S** | **I** | **R** | **S** | **I** | **R** |  |
| CIP | 6 (85.7%) | - | 1 (14.3%) | 1 (100%) | - | - | 5 (83.3%) | - | 1 (16.7%) | 1.000 |
| ERY | 1 (14.3%) | - | 6 (85.7%) | - | - | 1 (100%) | 1 (16.7%) | - | 5 (83.3%) | 1.000 |
| AZY | 1 (14.3%) | - | 6 (85.7%) | - | - | 1 (100%) | 1 (16.7%) | - | 5 (83.3%) | 1.000 |
| STX | 1 (14.3%) | 2 (28.6%) | 4 (57.1%) | - | 1 (100%) | - | 1 (16.7%) | 1 (16.7%) | 4 (66.6%) | 0.429 |
| GEN | 7 (100%) | - | - | 1 (100%) | - | - | 6 (100%) | - | - | 1.000 |
| TE | 1 (14.3%) | - | 6 (85.7%) | - | - | 1 (100%) | 1 (16.7%) | - | 5 (83.3%) | 1.000 |

**Table 14.** Multidrug resistance of *Campylobacter* isolates in HIV infected patients with viral load above and below 1000 copies/ml, with results of Fisher’s Exact test for differences.

| **Variable** | **Total Study n(%)** | | **>1000 copies/ml n(%)** | | **<1000 copies/ml n(%)** | | **P-value** |
| --- | --- | --- | --- | --- | --- | --- | --- |
|  | (n=7) | | (n=1) | | (n=6) | |  |
| MDR | **Yes** | **No** | **Yes** | **No** | **Yes** | **No** | 1.000 |
|  | 5 (71.4%) | 2 (28.6%) | 1 (100%) | - | 4 (66.7%) | 2 (33.3%) |  |

**Table 15.** Assessment of the association between *Campylobacter* antibiotic susceptibility profiles and HIV viral load in infected patients using Kruskal-Wallis test.

| **Variable** | **Categories** | **n** | **HIV Viral load** | | | | **Kruskal-Wallis H** | **P-value** |
| --- | --- | --- | --- | --- | --- | --- | --- | --- |
|  |  |  | **Average** | **SD** | **Min** | **Max** |  |  |
| CIP | S | 6 | 13,707.3 | 33,456.5 | 20 | 82,000.0 | 0.685 | 0.408 |
|  | I | - | - | - | - | - |  |  |
|  | R | 1 | 20 | N/A | 20 | 20 |  |  |
| ERY | S | 1 | 109 | N/A | 109 | 109 | 1.217 | 0.270 |
|  | I | - | - | - | - | - |  |  |
|  | R | 6 | 13,692.5 | 33,463.7 | 20 | 82,000.0 |  |  |
| AZY | S | 1 | 109 | N/A | 109 | 109 | 1.217 | 0.270 |
|  | I | - | - | - | - | - |  |  |
|  | R | 6 | 13,692.5 | 33,463.7 | 20 | 82,000.0 |  |  |
| STX | S | 1 | 109 | N/A | 109 | 109 | 2.136 | 0.344 |
|  | I | 2 | 41,010.0 | 57,968.6 | 20 | 82,000.0 |  |  |
|  | R | 4 | 33.8 | 27.5 | 20 | 75 |  |  |
| GEN | S | 7 | 11,752.0 | 30,976.5 | 20 | 82,000.0 | - | - |
|  | I | - | - | - | - | - |  |  |
|  | R | - | - | - | - | - |  |  |
| TE | S | 1 | 75 | - | 75 | 75 | 0.304 | 0.581 |
|  | I | - | - | - | - | - |  |  |
|  | R | 6 | 13,698.2 | 33,460.9 | 20 | 82,000,0 |  |  |
| MDR | Yes | 5 | 16,416.0 | 36,662.6 | 20 | 82,000.0 | 1.644 | 0.200 |
|  | No | 2 | 92 | 24.0 | 75 | 109 |  |  |
